# Solution-Processed High-k HfO_2_ Gate Insulator for High-Performance Indium-Zinc-Oxide Thin-Film Transistors: Optimisation of Annealing Temperature and Insulator Thickness

**DOI:** 10.3390/ma19101954

**Published:** 2026-05-09

**Authors:** Jialeen Sairike, Kamale Tuokedaerhan, Serikbek Sailanbek, Zhengang Cai, Haotian Yang

**Affiliations:** 1 School of Materials Science and Engineering, Xinjiang University, Urumqi 830046, China; hitjalen@163.com (J.S.);; 2 Educational Laboratory of Semiconductor Device Engineering, Faculty of Physics and Technology, Al-Farabi Kazakh National University, Almaty 050040, Kazakhstan

**Keywords:** HIGH-K, thin film transistor, insulation layer

## Abstract

**Highlights:**

- Systematic elucidation of annealing-temperature-dependent evolution of oxygen vacancies, M–O network densification, and crystallisation behaviour in sol–gel HfO_2_, directly correlating with TFT electrical performance and band alignment.
- All-solution-processed HfO_2_ high-K gate insulator and IZO active layer are achieved via the sol–gel method, eliminating expensive vacuum equipment.
- The optimised 85 nm HfO_2_ insulator annealed at 400 °C delivers outstanding device metrics (I_on/off_ = 1.11 × 10^6^, SS = 0.53 V/dec, Vth = −1.1 V), proving that solution-derived HfO_2_ is a viable and scalable alternative to conventional SiO_2_ for TFT miniaturisation without performance trade-off.

**Abstract:**

With the continuous advancement of display technology and advanced integrated circuits, oxide thin-film transistors (TFTs) have become core devices due to their high mobility, low leakage current and excellent large-area uniformity. To achieve low power consumption, high performance and high reliability, the introduction of high-k gate insulating layers is crucial. Among the numerous high-k materials, hafnium oxide (HfO_2_) has attracted significant attention due to its excellent dielectric properties and good compatibility with CMOS processes. In this paper, uniform and dense HfO_2_ films were successfully fabricated using the sol–gel method to serve as insulating layers for TFT devices. Through experimental analysis, 400 °C was determined to be the optimal annealing temperature. At this temperature, the effects of replacing SiO_2_ with HfO_2_ as the insulating layer, as well as the impact of reducing film thickness, on TFT devices were investigated. Ultimately, at an annealing temperature of 400 °C, an 85 nm-thick HfO_2_ film achieved the highest on/off current ratio (I_on/off_ = 1.11 × 10^6^), the lowest subthreshold swing (SS = 0.53 V/dec), the lowest threshold voltage (Vth = −1.1 V) and the lowest off-current ratio (I_off_ = 2.5 × 10^−12^ A). It was confirmed that replacing SiO_2_ with HfO_2_ as the insulating layer is a viable approach for reducing the volume of TFT devices.

## 1. Introduction

As the information age advances, integrated circuits have become a key indicator of high-end manufacturing capabilities. However, the ongoing pursuit of higher integration is encountering significant physical scaling limitations: as feature sizes enter the nanometre scale, issues such as short-channel effects, quantum tunnelling, leakage power consumption and thermal effects are becoming increasingly severe, and traditional silicon-based planar structures are approaching their physical limits in terms of performance and energy efficiency. Relying solely on proportional scaling not only makes it difficult to continue keeping pace with Moore’s Law, but may also lead to a decline in device reliability and a rise in manufacturing costs [1,2]. Consequently, overcoming scaling limitations urgently requires the introduction of novel channel materials (such as two-dimensional semiconductors and wide-bandgap materials) and innovative device structures (such as wrapped-gate and negative-capacitance transistors). These approaches are key to achieving the synergistic development of high performance and compact size, and they constitute the practical driving force behind this research [3,4,5].

In the scaling challenges described above, the physical limitations of the insulating layer are particularly pronounced. As the integration density of integrated circuits continues to increase, their physical size and scale also grow accordingly. Reducing the size of semiconductor devices has become a pressing challenge for researchers. Conventional SiO_2_ used as an insulating layer in semiconductor devices has yielded scant results in terms of miniaturisation due to its low bandgap and dielectric constant [6,7]. Consequently, researchers have explored the use of high-k dielectric materials to replace SiO_2_ as the insulating layer in semiconductor devices; however, most research in this area has been limited to the fabrication of MOS devices using high-k materials, with relatively few studies focusing on the integration of active layers and insulating layers to fabricate TFT devices [8,9].

In the field of thin-film transistors (TFTs), the use of high-k materials to replace conventional SiO_2_ has become an inevitable trend in order to reduce device operating voltage, enhance drive capability, and simultaneously suppress gate leakage current [10,11]. By comparing some of the mainstream high-k materials [12], as shown in Table 1, we can select a suitable one to serve as the insulating layer material.

Due to its high dielectric constant (k~20–25), wide bandgap (~5.7 eV), good thermal stability, and its potential to form high-quality interfaces with oxide semiconductor channels such as indium gallium zinc oxide (IGZO) and indium zinc oxide (IZO) has made it one of the most extensively studied high-k gate dielectric materials [13]. Recent research has not only focused on optimising the fabrication processes of HfO_2_ insulating layers to enhance the performance and stability of conventional TFTs, but has also explored the application of Hf-based materials in emerging fields such as ferroelectric field-effect transistors (FeFETs) [14,15].

The parameters of the HfO_2_ fabrication process (particularly atomic layer deposition, ALD) have a decisive influence on device performance. For example, Na et al. [16] found that in top-gate IGZO TFTs, increasing the temperature during ALD deposition of HfO_2_ (from 200 °C to 250 °C) effectively reduces the oxygen vacancy concentration in the channel layer, thereby significantly improving the device’s stability under negative bias-induced stress (NBIS), with Vth drift decreasing from −3.6 V to −2.4 V. This indicates that high-temperature ALD facilitates the formation of denser, defect-poorer HfO_2_ films and reduces damage to the underlying oxide channel. Furthermore, the introduction of N_2_O plasma treatment or specific annealing processes following HfO_2_ deposition can also effectively passivate interface states, thereby enhancing device performance. Liu et al. [17] fabricated Hf-doped IZO TFTs via PEALD, combined with an HfO_2_ gate dielectric, achieving a high mobility of 21.7 cm^2^/Vs and an ultra-low SS of 69 mV/dec.

Due to its strong affinity for oxygen, Hf exhibits two distinct functional mechanisms in semiconductor devices. The first is its role as an oxygen adsorption/removal layer in thin-film transistors (TFTs), where it regulates the distribution of oxygen vacancies within the channel. Kim et al. [18] introduced Hf-doped IGZO (IGZO:Hf) into IGZO TFTs as an “oxygen adsorption layer”; During annealing, this layer absorbs oxygen ions from the main channel layer (a-IGZO), thereby generating more oxygen vacancies in the main channel to enhance mobility, whilst forming fewer oxygen vacancies in the back channel to improve stability, resulting in an increase in mobility from 9.25 cm^2^/Vs to 31.08 cm^2^/Vs. Wang et al. [19] also proposed a similar double-layer channel structure (indium zinc oxide IZO/hafnium indium zinc oxide HIZO), utilising Hf’s adsorption of oxygen to achieve a vertical gradient distribution of oxygen vacancies, whilst simultaneously enhancing mobility (7.08 cm^2^/Vs) and bias/illumination stability. Secondly, in contrast to the aforementioned oxygen adsorption mechanism, the emergence of HfO_2_-based ferroelectric materials (such as hafnium-zirconium oxide, HfZrO_x_) has endowed gate insulating layers with non-volatile storage capabilities [20]: By doping HfO_2_ with elements such as Zr and Si and subjecting it to specific annealing, a ferroelectric orthorhombic phase (o-phase) can be induced. Using this ferroelectric material as the gate insulator enables the fabrication of ferroelectric field-effect transistors (FeFETs), thereby achieving non-volatile data storage.

Although the aforementioned research has yielded remarkable results, there remain significant limitations in terms of advancing low-cost, large-area, and flexible electronic technologies. For instance, the high fabrication costs and process complexity are notable issues; the preparation of most high-performance Hf-based insulating layers relies on vacuum equipment such as ALD and sputtering [21]. These devices are expensive, involve complex processes, and typically require high-temperature treatment (>400 °C) [22,23], which not only increases manufacturing costs but also results in poor compatibility with flexible plastic substrates [24].

In contrast, there has been relatively little research into the preparation of Hf-based insulating layers using low-cost, non-vacuum, and easily scalable chemical solution methods such as the sol–gel process. Although a few attempts, such as the HfAlOx-PVP hybrid dielectric layer [25], have demonstrated the potential of low-temperature solution processes, there is still significant room for improvement in terms of mobility (~0.25 cm^2^/Vs) and electrical performance. To quantify the competitiveness of the sol–gel method, we conducted a quantitative comparison of the dielectric constant of HfO_2_ films prepared by the sol–gel method with an ALD reference. Blanchin et al. [26] employed the sol–gel method to prepare multilayer HfO_2_ films under annealing conditions of 400–600 °C. Capacitance measurement results indicate that the dielectric constant reached 25, which is very close to the value for HfO_2_ material (approximately 25). This result clearly demonstrates that the sol–gel method is fully capable of producing HfO_2_ insulating layers with a dielectric constant close to the theoretical value. As a representative of vacuum-based methods, HfO_2_ films deposited by ALD typically have a dielectric constant in the range of 20–25. Park et al. [27] employed a novel iodinated Hf precursor to prepare HfO_2_ films via the ALD process, achieving an ultra-low leakage current density at extremely thin thicknesses, representing the current state-of-the-art in vacuum-based HfO_2_ insulation layer fabrication, with a leakage current density as low as 7.02 × 10^−8^ A/cm^2^ at an equivalent oxide thickness (EOT) of 1.73 nm. Furthermore, Kim et al. [28] used plasma-enhanced atomic layer deposition (PEALD) to fabricate HfO_2_ films at a low temperature of 80 °C, achieving a dielectric constant of approximately 20 and a leakage current density of 2.5 × 10^−5^ A/cm^2^.

The above quantitative comparison indicates that, whilst the ALD process offers significant advantages in leakage control at the ultra-thin scale (EOT < 2 nm), the sol–gel method is fully capable of achieving a dielectric constant comparable to that of the ALD method (ε ≈ 20–25). More importantly, the sol–gel method does not require vacuum equipment and can be carried out under atmospheric conditions, significantly reducing manufacturing costs and process barriers, making it particularly suitable for large-area and flexible electronic applications, and even for use in the medical field. For example, Sabbagh et al. [29] incorporated MgZnO nanoparticles prepared by the sol–gel method into a κ-carrageenan/PVA hydrogel and found that an increase in ZnO content could improve surface roughness, mechanical properties, and the encapsulation and release rates of catechins (with 5% ZnO yielding the best results); furthermore, the material was non-cytotoxic, providing a reference for the design of functional wound dressings. Therefore, although performance gaps exist at the ultra-thin scale, the all-solution method demonstrated in this work offers irreplaceable advantages in terms of cost-effectiveness and process simplicity. Furthermore, research into the integration of solution-processed Hf-based insulating layers with high-mobility channel materials (such as IZO), which are also prepared via solution processes, remains insufficient.

Based on the above analysis, this paper proposes the fabrication of TFTs combining Hf-based insulating layers with IZO active layers using a fully solution-based process, with the aim of developing a TFT device that combines high performance, low cost and process simplicity [30]. The core strengths and innovations of this study are reflected in the following aspects:

Firstly, the all-solution process offers significant cost-effectiveness. In this work, both the insulating layer and the active layer are prepared using the sol–gel method, completely eliminating the need for expensive vacuum equipment [9]. This greatly reduces production costs and process complexity, providing a highly competitive technical solution for the future manufacture of large-area, flexible electronic devices.

Secondly, performance optimisation and mechanism exploration: by systematically controlling process parameters such as the composition of Hf-based precursors and annealing temperature [9], this study will delve deeply into the intrinsic relationship between the composition, structure and electrical properties of Hf-based insulating layers. Combined with systematic characterisation of the IZO/Hf-based insulating layer interface [31,32,33], we will elucidate the physical mechanisms influencing the electrical performance and stability of TFTs, thereby providing experimental evidence and theoretical guidance for the rational design of solution-processed Hf-based insulating layers.

In summary, this study aims to fill this gap. By fabricating all-solution-processed, high-performance, and highly stable Hf-based insulating layer/IZO active layer TFTs, we will not only provide a viable technical pathway for low-cost, large-area electronic devices but also, through in-depth analysis of physical properties, enrich our understanding of solution-processed oxide interfaces and drive further development in this field.

## 2. Materials and Methods

First, the active layer precursor solution is prepared by dissolving 0.1 M zinc nitrate hexahydrate (Zn(NO_3_)_2_·6H_2_O, Aladdin, Shanghai, China, 99%) and 0.1 M indium nitrate hydrate (In(NO_3_)_3_·xH_2_O, Aladdin, Shanghai, China, 99%) in a 1:1 ratio in 20 mL of diethylene glycol methyl ether solvent. The mixture is then placed in a digital temperature-controlled magnetic stirring water bath and stirred at an appropriate speed for 16 h at 50 °C.

Next, the solution of the HfO_2_ precursor for the insulating layer was prepared, using hafnium tetrachloride (HfCl_4_, Aladdin, Shanghai, China, 99%) and diethylene glycol methyl ether (C_3_H_8_O_2_, Aladdin, Shanghai, China, 99%) as the precursor and solvent, respectively. Molar ratio: A solution with a concentration of 0.1 mol/L was prepared by dissolving the precursor in 20 mL of diethylene glycol methyl ether. The calculated molar ratio of HfCl_4_ to the solvent was approximately 1:107 (density of diethylene glycol methyl ether: 0.965 g/mL; molecular weight: 90.12; 20 mL corresponds to approximately 0.214 mol). Place the mixture in a digital temperature-controlled magnetic stirring water bath, set the temperature to 50 °C, and stir for 16 h to promote the hydrolysis and condensation reaction of the precursor in the solvent. Upon completion of stirring, the resulting solution was transferred to plastic centrifuge tubes, sealed and stored, and aged for 48 h at room temperature (25 ± 2 °C) and a relative humidity of 35–45%, yielding a clear, transparent HfO_2_ precursor solution. During the preparation process, hydrolysis/condensation reactions occur in the solution, as shown in Equations (1) and (2) [34]:

(1) $$M-OH+HO-M\to M-O-M+H_{2}O$$

(2) M−OR+HO−M→M−O−M+ROH

**Figure 1:**
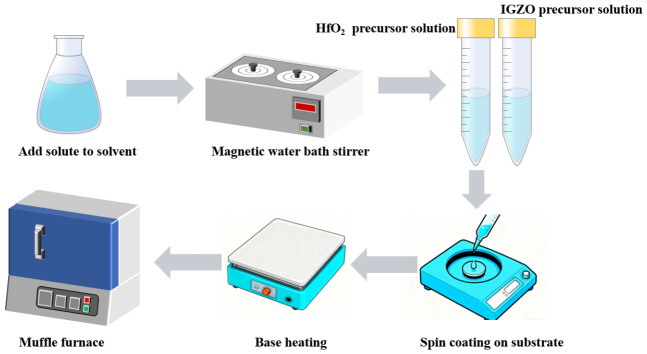
Sample preparation.

Finally, following a post-processing heat treatment, a large proportion of the liquid phase in the precursor solution is removed, ultimately transforming the gel into a film. The process is shown in Figure 1.

In this work, n-Si (100) with a resistivity of 1–5 Ω·cm was used as the substrate. Prior to spin-coating the thin film onto the Si substrate, the substrate must be cleaned with reagents to remove any impurities on the surface that may affect device performance. The substrate was cleaned for 10 min in an ultrasonic cleaner using acetone, ethanol and deionised water, respectively. The aim is to remove organic contaminants, impurity ions and particulate impurities from the surface of the Si substrate, thereby minimising factors that affect device performance. Prior to cleaning with deionised water, the Si substrate must be ultrasonically cleaned for 10 min in a 50-fold diluted HF solution to remove the oxide layer from its surface. The cleaned silicon wafers are placed in a spin coater, and the insulating layer precursor solution is spin-coated onto the silicon substrate. All spin-coating processes are carried out at room temperature (22–25 °C) and a relative humidity of 40% ± 5%. The spin-coating parameters are fixed at 3000 rpm and 20 s. After each spin-coating layer, the wafer is pre-baked on a 200 °C hot plate for 10 min to remove residual solvent. By controlling the number of spin-coated layers (4–5 layers), HfO_2_ films of varying thicknesses (approximately 16–18 nm per layer) can be obtained. The thickness is measured by taking the average of three repeated measurements using an ellipsometer, ensuring that the thickness deviation between batches is less than 5%.

After spin-coating the insulating layer, the spin-coated silicon wafers were annealed in a rapid annealing furnace at various annealing temperatures. They were then placed back on the spin coater for the spin-coating of the IZO active layer. After four layers had been applied (~65 nm), the wafers were annealed again in the rapid annealing furnace at 350 °C to produce the final IZO/HfO_2_ TFT devices. Finally, the TFT electrodes (Al) are sputtered using the shadow mask method in a DC magnetron sputtering system, with an electrode thickness of approximately 30 nm. The electrode width is W = 100 μm, and the aspect ratio W/L is 1/10. The fabricated IZO/HfO_2_ TFT devices are then characterised in terms of structural properties, optical properties, surface morphology and electrical properties. Whether preparing the precursor solution or during the spin-coating process, environmental conditions such as temperature and humidity must be kept stable; otherwise, the performance of the resulting samples will deteriorate significantly, leading to a decline in quality. The prepared solution should be used as soon as possible (as it typically begins to degrade after 72 h); a degraded precursor solution will cause the samples to lose their electrical properties.

## 3. Results and Discussion

**Figure 2:**
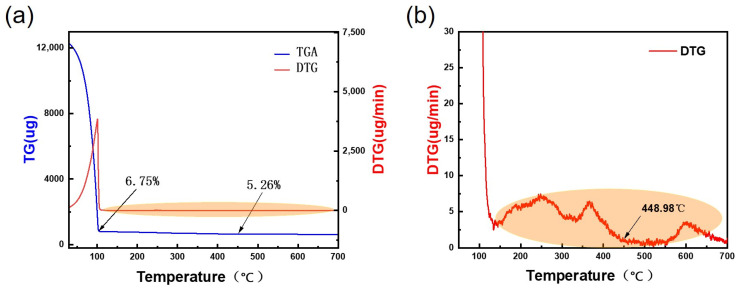
HfO_2_ solution: (**a**) DTA-TGA curve; (**b**) zoomed-in section of the DTG curve.

To gain a deeper understanding of the effect of temperature on HfO_2_, differential thermal analysis (DTA) and thermogravimetric analysis (TGA) were performed on the HfO_2_ precursor solution; the results are shown in Figure 2.

As can be seen from the TGA curve in Figure 2, as the temperature gradually rises to 100 °C, the HfO_2_ solution loses 93.25% of its weight during this process, which is attributed to the evaporation of a large amount of organic solvent. To prevent the solvent from adversely affecting the film as it evaporates, the spun-coated insulating layer should be heated on a hot plate at a temperature of 110 °C or higher for 5 min (200 °C was selected in this study) immediately after spin-coating, in order to remove the majority of the solvent. However, when the temperature was gradually raised to 450 °C, the weight loss was only 1.49% compared to that at 100 °C. This weight loss encompasses physically adsorbed water in the HfO_2_ solution, residual organic solvents, and chemical changes occurring within the HfO_2_ solution. As the changes were too subtle and there were too many variables, we performed a localised magnification analysis of the DTG curve. From Figure 2b, we can observe two peaks in the rate of weight change within the 100–450 °C range. Based on the analysis of the first peak, the former corresponds to the decomposition peak of Hf ions in the precursor. At this temperature, the metal separates from OH^−^ ions, and a small amount of water vapour is generated under the influence of temperature, whilst the remaining O^2−^ ions combine with Hf^4+^ ions as the temperature rises, forming an M-O-M metal network; this constitutes the second fluctuation peak. In nature, most chemical reactions do not occur in isolation; they often overlap in time. As the crystallisation temperature of HfO_2_ is relatively low, it cannot be ruled out that the formation of the M-O-M metal network within the film is accompanied by a crystallisation process. Both processes occur simultaneously within a certain temperature range. The annealing temperatures for the samples were therefore set at 350 °C, 400 °C, 450 °C and 500 °C. To verify whether the prepared solution was indeed an HfO_2_ precursor solution and to determine its crystallisation temperature in order to establish the specific annealing conditions, the prepared films were subjected to full-scan XPS analysis and XRD testing.

**Figure 3:**
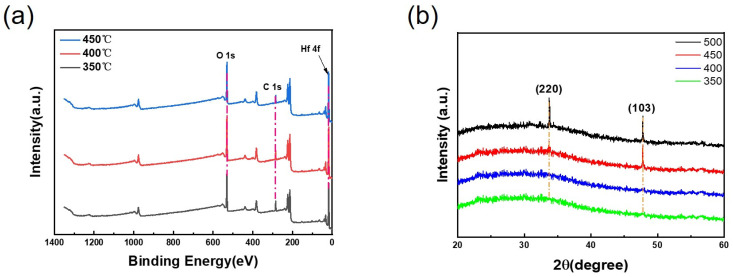
(**a**) XPS full spectrum; (**b**) XRD pattern.

As shown in Figure 3a, a full-scan XPS analysis was performed on the precursor solution. From the spectrum, the Hf 4f and O 1s peaks are clearly visible, indicating that the HfO_2_ precursor was successfully prepared using the sol–gel method. Figure 3b shows the data curves obtained from XRD analysis of HfO_2_ films after annealing at different temperatures. From the figure, it is clearly observed that crystallisation occurs in the HfO_2_ films at annealing temperatures of 450 °C and 500 °C. Crystallisation of HfO_2_ can be detected at 450 °C. In the weight loss rate graph shown in Figure 2b, we can see that the second peak is very close to the crystallisation temperature. However, the formation of a crystalline structure does not occur instantaneously; it typically involves a certain process, and the resulting crystalline structure is detected, thereby allowing us to determine that crystallisation has occurred. As crystalline structures form leakage channels in TFT devices, causing a significant negative impact on their electrical performance—which is undesirable in this study—XRD provides a highly valuable reference for determining the annealing temperature gradient of the devices.

During the sample preparation process, XRD analysis of the HfO_2_ film revealed that crystallisation occurred at 450 °C. Consequently, in order to prevent crystallisation and ensure thorough annealing, we conducted subsequent studies using annealing temperatures of 350 °C, 400 °C and 450 °C.

**Figure 4:**
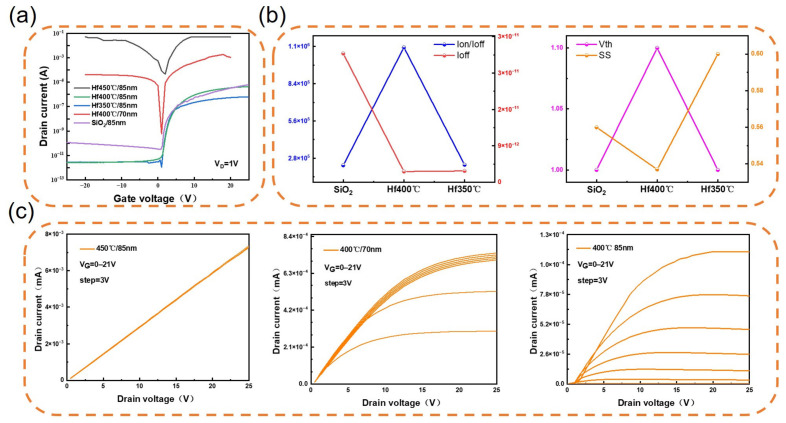
Electrical performance of IZO/HfO_2_ TFTs under different conditions: (**a**) I_D_-V_G_; (**b**) Vth, SS, I_off_ and I_on/off_; (**c**) I_D_-V_D_.

During the fabrication of IZO/HfO_2_ TFTs, the annealing temperature for the IZO active layer is 350 °C. Furthermore, XRD analysis of the HfO_2_ film revealed that crystallisation occurs at 450 °C; consequently, we ultimately established an annealing temperature gradient comprising 350 °C, 400 °C and 450 °C. As shown in Figure 4a, with the HfO_2_ insulating layer uniformly set at 85 nm thick, compared to TFT devices annealed at 350 °C and 400 °C, the leakage current of the IZO/HfO_2_ TFTs annealed at 450 °C was as high as 10^−1^ A. This result is attributed to the XRD findings shown in Figure 3b: at an annealing temperature of 450 °C, a crystalline structure forms within the HfO_2_ film. The presence of grain boundaries provides favourable pathways for current flow, resulting in excessive leakage current in the device; this conclusion is verified by Figure 4c.

As can be seen from Figure 4c, the current ID is in a perfectly linear relationship with the voltage VD, indicating a complete loss of the semiconductor’s switching characteristics, which renders the device completely unusable. In contrast, IZO/HfO_2_ TFTs annealed at 350 °C and 400 °C, with the same insulating layer thickness, exhibit complete semiconductor switching characteristics. We compared the electrical performance of these devices with that of IZO/SiO_2_ TFTs, and the comprehensive results are shown in Figure 4b.

In particular, the subthreshold swing (SS) is a key electrical parameter of TFT devices; its physical significance lies in the change in gate voltage (V_G_) when the drain current (I_D_) changes by an order of magnitude. A smaller SS indicates higher switching efficiency of the device. The expression is as follows [35]:

(3) $$SS=\frac{\Delta V_{G}}{\left|\Delta \log\left.I_{ds}\right|\right.}$$

Carrier mobility (μ) represents the average drift velocity of carriers in a uniform electric field; the higher the mobility, the greater the conductivity and the faster the switching between positive and negative voltages, which manifests as a higher refresh rate in electronic displays. The expression is as follows [36]:

(4) $$\mu =\frac{g_{m}L}{WV_{D}C_{ox}}$$

g_m_ is the transconductance (g_m_ = dI_D_/dV_G_). C_ox_ is the corresponding gate capacitance. The threshold voltage (Vth) under different conditions can also be determined from the curve.

From the figure, it can be seen that, at the same insulating layer thickness and an annealing temperature of 400 °C, the IZO/HfO_2_ TFT with an HfO_2_ insulating layer exhibits the best performance, featuring lower subthreshold swing (SS), Vth, and off-current (I_off_), as well as a higher on-off current ratio (I_on/off_). The specific values are shown in Table 1.

From Table 2, we conclude that, for the same insulating layer thickness, the electrical performance is optimal for an 85 nm HfO_2_ insulating layer annealed at 400 °C, exhibiting lower subthreshold swing and lower leakage current, and a higher on/off current ratio. This is primarily attributed to the fact that HfO_2_ possesses a higher dielectric constant than SiO_2_; its high dielectric constant means that a greater physical thickness (d) can be used to achieve the same capacitance value as that of an extremely thin SiO_2_ layer, a characteristic known as the equivalent oxide thickness (EOT). This demonstrates that replacing SiO_2_ with a high-k dielectric material as the insulating layer makes it feasible to reduce the volume of TFT devices by decreasing the film thickness whilst maintaining the same electrical performance.

Furthermore, as shown in Figure 4a, at an annealing temperature of 400 °C, the switching performance of the device deteriorates to some extent as the insulating layer thickness is further reduced. The primary cause of this phenomenon is not solely due to limitations imposed by the bandgap width (E_g_), but is related to the significant enhancement of defect-assisted tunnelling effects in thinner insulating layers. When the insulating layer is thinned, even if a high E_g_ is maintained, defects such as trap states and oxygen vacancies within the film and at the interfaces can still provide tunnelling pathways for carriers, leading to an increase in leakage current. Concurrently, the increase in interface state density also weakens the gate’s ability to effectively modulate the channel. Consequently, the degradation in switching performance is primarily driven by defect-induced leakage mechanisms, rather than being determined solely by the magnitude of E_g_.

**Figure 5:**
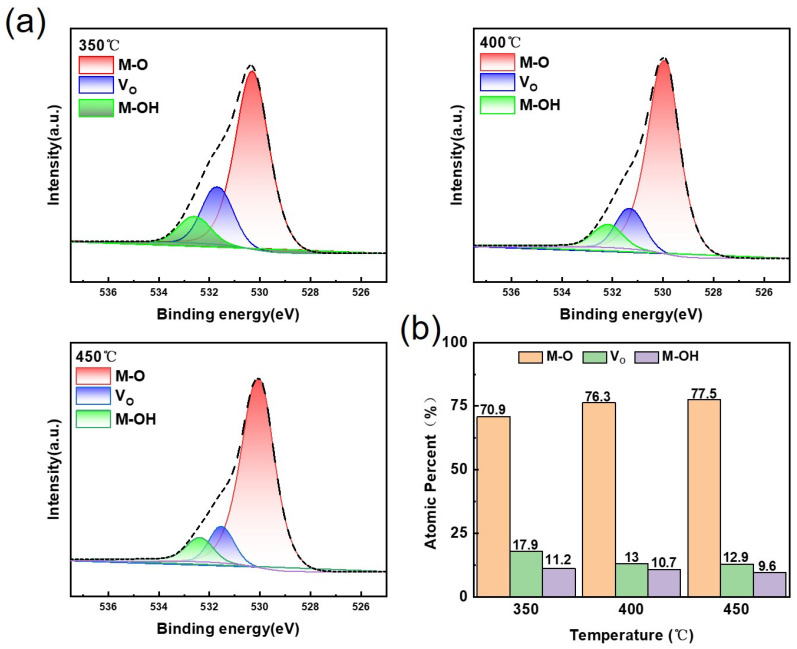
(**a**) O 1s energy spectrum; (**b**) proportion of oxygen vacancies at different annealing temperatures.

Following peak splitting and fitting of the O 1s peak in Figure 5a, three sub-peaks were identified, with peak centres at 530.3 eV, 531.4 eV and 532.5 eV, respectively. These can be attributed to the metal-oxygen (M-O) bond, oxygen vacancies (V_O_), and the contribution of hydroxyl (OH^−^) or adsorbed oxygen, in that order. It should be noted that the results of XPS peak fitting are highly dependent on fitting parameters (such as peak shape, full width at half maximum, background subtraction method and peak position constraints); therefore, the proportion of oxygen vacancies derived from the fitted area ratio should be regarded as a semi-quantitative analysis result rather than an absolutely accurate value. Under the fitting conditions employed in this study, the proportion of oxygen vacancies exhibits a decreasing trend with increasing annealing temperature: following annealing at 350 °C, 400 °C and 450 °C, the relative proportions of oxygen vacancies obtained from the fitting were 17.9%, 13.0% and 12.9%, respectively. This trend indicates that increasing the annealing temperature promotes the formation of M-O bonds and reduces oxygen vacancies, thereby improving the film’s performance.

**Figure 6:**
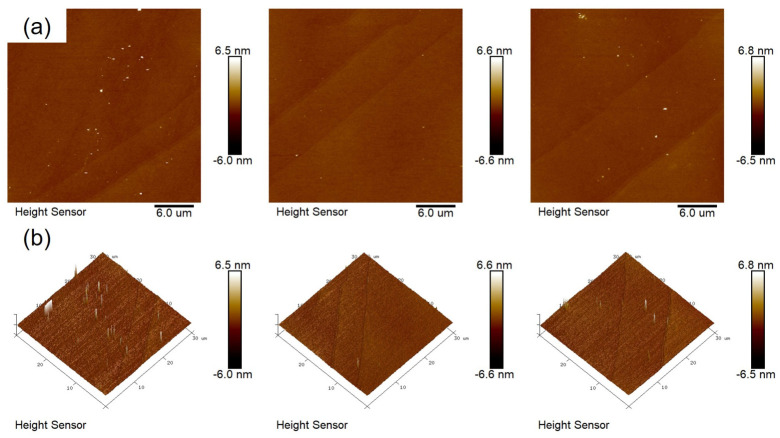
(**a**) A 2D AFM image; (**b**) 3D AFM image at different annealing temperatures.

Figure 6 shows atomic force microscopy (AFM) images of films annealed at 350 °C, 400 °C and 450 °C to analyse the effect of different annealing temperatures on the surface topography of the films. Figure 6a shows a 2D image of the film surface; from left to right, the root-mean-square (RMS) roughness of the films was measured as 0.264 nm, 0.130 nm and 0.144 nm, respectively. The AFM results show that the surface of the sample annealed at 350 °C exhibits significant topographical variations and a high root-mean-square (RMS) roughness. This is consistent with the trend of a higher oxygen vacancy fraction observed in the semi-quantitative XPS analysis, indicating that the film is insufficiently densified at lower annealing temperatures and contains a higher number of defects. However, it should be noted that there is no direct causal relationship between AFM roughness and the oxygen vacancy ratio; the two reflect the film’s morphological characteristics and chemical bonding information, respectively. The correlation in their trends collectively supports the overall conclusion that higher annealing temperatures are beneficial for improving film quality. Conversely, the sample annealed at 400 °C exhibits the lowest RMS value. This is because, compared to the film annealed at 350 °C, the former has a lower proportion of oxygen vacancies, and the elevated temperature facilitates the formation of M-O bonds, resulting in a more compact oxide film. At an annealing temperature of 450 °C, however, as the temperature increased, the film exhibited some fluctuations and the RMS value rose slightly. This was due to crystallisation occurring in the film at this temperature, resulting in the appearance of crystalline particles on the surface; this finding is corroborated by the conclusions drawn from the XRD analysis in Figure 3b. Therefore, at an annealing temperature of 400 °C, the surface topography of the film exhibits the lowest RMS value and the best topographical performance.

**Figure 7:**
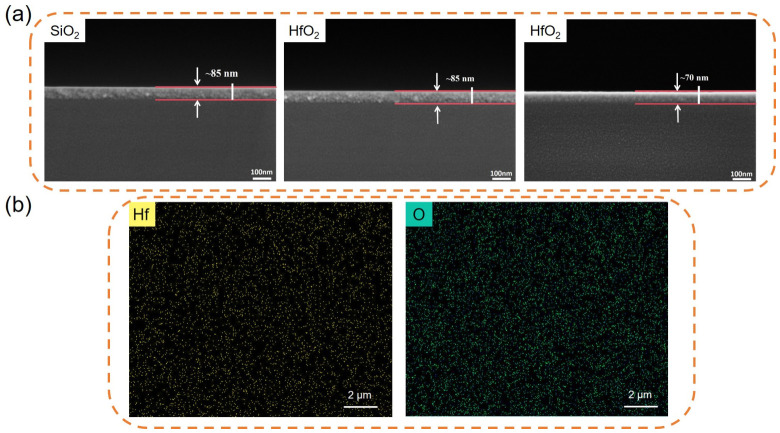
(**a**) SEM images of the device insulator surface and cross-section; (**b**) SEM mapping of an 85 nm HfO_2_ insulator annealed at 400 °C.

Figure 7a shows SEM images of the surface and cross-section of the SiO_2_ insulating layer in the first image, whilst the latter two images depict SEM images of the surface and cross-section of HfO_2_ insulating layers of varying thicknesses. For the HfO_2_ insulating layers of different thicknesses, we prepared them by varying the number of spin-coating cycles whilst maintaining the same spin-coating speed and duration during the film preparation process. We dissolved 1 mL of HF in 50 mL of deionised water and stirred thoroughly until the HF was completely diluted in the deionised water. The SiO_2_ substrate was then placed in the diluted HF solution, left to stand for 30 s, then rapidly removed and placed in anhydrous ethanol. After rinsing off the HF, the SiO_2_ substrate was placed on a heating stage to evaporate the anhydrous ethanol before spin-coating. It should be noted that this experiment did not include a control sample that had not undergone HF treatment; consequently, the actual impact of this process on interface quality and the final device performance has not been fully demonstrated here. This step is adopted from the standard process and is included solely as part of the routine procedure.

As shown in Figure 7, following annealing at 400 °C, the surfaces of all three films were smooth and free of cracks or defects, with thicknesses of approximately 85 nm, 85 nm and 70 nm, respectively. Figure 7b shows SEM mapping of the 85 nm HfO_2_ insulating layer following annealing at 400 °C. The figure reveals that Hf and O elements are uniformly distributed within the film, indicating good film uniformity; this fully explains the results presented in Figure 6.

The bandgap Eg at different annealing temperatures can be fitted using a linear extrapolation method based on the Tauc formula. The Tauc relationship is as follows [37]:

(5) $$\sqrt{\left(\alpha hv\right)}=A\left(hv-E_{g}\right)$$

**Figure 8:**
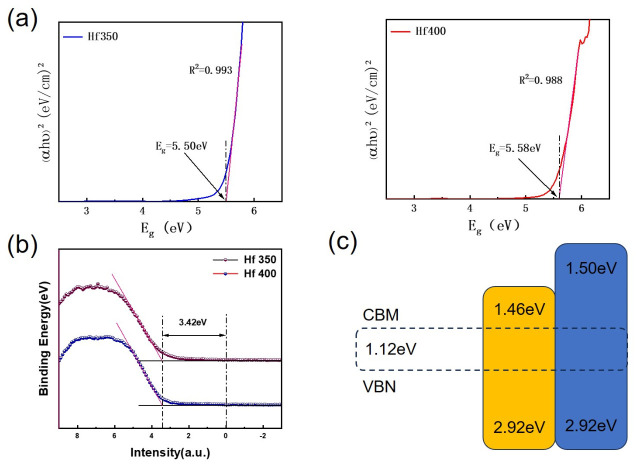
(**a**) Bandgap Eg; (**b**) valence band energy EV; (**c**) schematic diagram of the energy bands at different annealing temperatures.

Among them, α is the molar absorption coefficient, h is the Planck constant, v is the incident photon frequency, A is the proportional constant, and E_g_ is the optical bandgap of the semiconductor material. The bandgap (E_g_) at different annealing temperatures can be fitted using linear extrapolation based on the Tauc equation. The coefficients of determination (R^2^) for the samples annealed at 350 °C and 400 °C were 0.993 and 0.988, respectively, indicating that linear extrapolation is highly reliable. The fitted E_g_ values were 5.50 eV (350 °C) and 5.58 eV (400 °C), as shown in Figure 8a. Figure 8b presents the valence band data for HfO_2_ at different annealing temperatures; under these temperature variations, the valence band remains essentially unchanged. The change in E_g_ is due to the fact that the E_g_ data were fitted from the optical bandgap, which depends on the film’s transmittance; however, as the change is very slight (only 0.08 eV), only a general trend of the bandgap increasing with rising temperature can be observed. Figure 8c is a schematic diagram of the HfO_2_ band structure. The valence band offset (ΔE_V_) at the interface between HfO_2_ and the Si substrate under different thermal treatment conditions can be estimated using the valence band maximum (VBM). The valence band offset of the HfO_2_/Si gate stack can be determined by measuring the maximum valence band energy (VBM) between the HfO_2_ film and the Si substrate, which is given by the following equation [38]:

(6) $$∆E_{V}\left({HfO}_{2}/Si\right)=E_{V}\left(HfO_{2}\right)-E_{V}\left(Si\right)$$

Here, E_V_(Si) = 0.50 eV. Using the above equation, the valence band offset for samples annealed at 350 °C and 400 °C can be calculated as 2.92 eV. For TFT devices, the minimum barrier height of the conduction band offset of the film must reach 1 eV; this is required to block the transport of electrons and holes during device fabrication. Using the bandgap of the high-k film and the ΔE_V_ between the film and the Si substrate, the conduction band offset (ΔE_C_) can be derived from the following equation [38]:

(7) $${∆E}_{C}\left(HfO_{2}/Si\right)=E_{g}\left(HfO_{2}\right)-{∆E}_{V}\left(HfO_{2}/Si\right)-E_{g}\left(Si\right)$$

The E_g_ (HfO_2_) and E_g_ (Si) are the band gaps of the HfO_2_ film and Si, respectively. The value of E_g_ (Si) is 1.12 eV. The resulting conduction band offsets (ΔE_c_) for the film annealed at 350 °C and 400 °C were calculated to be 1.46 eV and 1.50 eV, respectively. By comparison, the conduction band offset at the HfO_2_/Si interface measured by XPS/UPS in the literature typically falls within the range of 1.4–1.8 eV; for example, Li et al. [38] reported a ΔE_c_ of approximately 1.5 eV for sol–gel HfGdO films, whilst that for ALD-HfO_2_/Si was approximately 1.5–1.7 eV. The ΔE_c_ values obtained in this work (1.46–1.50 eV) are in close agreement with the values reported in the literature, indicating that HfO_2_ films prepared by the sol–gel method possess band alignment characteristics comparable to those of materials prepared by vacuum deposition, thereby effectively suppressing carrier injection leakage current.

As the annealing temperature increases, the conduction band offset ΔE_C_(HfO_2_/Si) increases. This means that the process by which carriers and holes transition from the valence band to the conduction band requires more energy, giving the TFT device greater control over carriers and holes. This facilitates lower leakage current and a higher on-off current ratio, which provides a reasonable explanation for the electrical data shown in Figure 4: under the same insulating layer thickness, TFT devices fabricated at an annealing temperature of 400 °C exhibit the best electrical performance. The energy gap (E_g_) of HfO_2_ is 5.58 eV, whereas SiO_2_ appears too weak in comparison to high-K materials. This provides a reasonable explanation for the increase in leakage current observed when reducing the film thickness in devices using SiO_2_ as the insulating layer; this is because SiO_2_ has a lower dielectric constant, resulting in weaker control over carriers and holes within the device. At the same physical thickness, the lower dielectric constant of SiO_2_ leads to a larger equivalent oxide thickness (EOT); When SiO_2_ is thinned to achieve high capacitance, direct tunnelling and defect-assisted tunnelling become the dominant leakage mechanisms. HfO_2_, however, with its high dielectric constant, can achieve the same capacitance value at a greater physical thickness, thereby effectively suppressing tunnelling leakage. Furthermore, defect-related factors such as the interface state density and oxygen vacancy concentration at the HfO_2_–IZO channel interface often exert a more significant influence on leakage current and switching characteristics than the simple difference in E_g_ alone. Therefore, when discussing leakage current, one should comprehensively consider defect-assisted tunnelling, interface trap density, and the actual impact of the dielectric constant on the effective electrical thickness. Similarly, replacing SiO_2_ with HfO_2_—a high-k dielectric material with a lower bandgap—as the insulating layer is an effective means of reducing the TFT volume without compromising electrical performance; furthermore, TFT devices fabricated with IZO as the active layer and HfO_2_ as the insulating layer have demonstrated excellent electrical performance.

## 4. Conclusions

In this study, uniform and dense HfO_2_ films were successfully fabricated using the sol–gel method to replace SiO_2_ as the insulating layer in TFT devices. Through a detailed analysis of the changes in the precursor solution as the temperature increased, a scientifically sound annealing temperature gradient was determined for the HfO_2_ films. The effect of annealing temperature on the electrical performance of TFT devices was investigated for films of the same thickness. After determining 400 °C to be the optimal annealing temperature, the effects of using HfO_2_ in place of SiO_2_ as the insulating layer and of reducing the film thickness on TFT devices were studied at this annealing temperature. Various characterisation tests were performed on the films, and the results of the electrical tests were analysed in detail in conjunction with the characterisation data. Ultimately, at an annealing temperature of 400 °C, an 85 nm thick HfO_2_ layer exhibited the optimal electrical performance, with I_off_ of 2.55 × 10^−12^ A, Vth of 1.1 V, SS of 0.53 V/dec, μ of 49.8 and I_on/off_ of 1.11 × 10^6^. Under the device structures and process conditions studied, replacing SiO_2_ with HfO_2_ as the insulating layer is a viable method for reducing the size of TFT devices.

Furthermore, to explore the feasibility of extending IZO/HfO_2_ TFTs fabricated via this all-solution process to flexible electronic devices, future research requires a systematic evaluation of the devices’ long-term bias stress stability (e.g., positive bias stress (PBS) and negative bias illumination stress (NBIS)) as well as mechanical flexibility (e.g., electrical performance degradation and cyclic bending fatigue life under different bending radii). This work provides the fundamental device structures and process parameters for the aforementioned research. In the future, through the optimisation of interface engineering and encapsulation strategies, it is expected that low-cost, highly stable TFT devices suitable for applications such as flexible displays and wearable devices can be realised.

## Figures and Tables

**Table 1:** Dielectric constants and bandgap values of different materials.

Insulation Layerl	Dielectric Constant	Bandgap
HfO_2_	25	5.3~5.9
Al_2_O_3_	10	8.7
TiO_2_	80	3.5
Ta_2_O_5_	22	4.4

**Table 2:** Electrical properties of TFTs at different annealing temperatures for the same thickness.

	Vth (V)	SS (V/dec)	μ	I_off_ (A)	I_on/off_
400 °C 85 nm (SiO_2_)	1	0.56	13.2	3.07 × 10^−11^	2.26 × 10^5^
400 °C 85 nm (Hf)	1.1	0.53	49.8	2.55 × 10^−12^	1.11 × 10^6^
350 °C 85 nm (Hf)	1	0.60	46.8	2.70 × 10^−12^	2.30 × 10^5^

## Data Availability

The original contributions presented in this study are included in the article. Further inquiries can be directed to the corresponding author.
